# The species of *Haplothrips* (Thysanoptera, Phlaeothripinae) and related genera recorded from the Hawaiian Islands

**DOI:** 10.3897/zookeys.662.12107

**Published:** 2017-03-21

**Authors:** Laurence A. Mound, Janis N. Matsunaga

**Affiliations:** 1 CSIRO Australian National Insect Collection, PO Box 1700, ACT 2601, Australia; 2 Hawaii Department of Agriculture, Plant Pest Control Branch, 1428 S. King St., Honolulu, Hawaii, USA

**Keywords:** Endemics, introduced species, Haplothripini, mycophagy

## Abstract

An illustrated identification key is provided to 17 species of Thysanoptera: Phlaeothripinae from Hawaii that are members of the Tribe Haplothripini, together with a further species that is similar in general appearance to members of that Tribe. Of these 18 species, 13 are considered introduced from other parts of the world, but five appear to be endemics. Known only from Hawaii, *Haplothrips fissus* **syn. n.** is considered to have been based on a teratological specimen and is placed as a synonym of the Hawaiian endemic *Haplothrips davisi*. Both this species and two further endemics, *Haplothrips rosai* and *Priesneria doliicornis*, are possibly mycophagous rather than phytophagous. The Indonesian species *Haplothrips sesuvii* **syn. n.** is recognised as a synonym of *Haplothrips robustus* from Australia, although both names have been used in Hawaii. Two further species that are presumed to be Hawaiian endemics, *Apterygothrips remotus* and *Haplothrips williamsi*, remain known only from the original specimens.

## Introduction

The Tribe Haplothripini constitutes one of three major lineages within the subfamily Phlaeothripinae (Mound and Minaei 2007), and *Haplothrips* is by far the largest genus in this group, with 243 species listed worldwide (ThripsWiki 2017). Most *Haplothrips* species breed in flowers, and the recorded hosts involve a wide range of genera, particularly in the families Asteraceae and Poaceae. In contrast, some species of Haplothripini are predatory, and one or more species endemic to Hawaii are possibly mycophagous. The information presented here concerning Haplothripini recorded from the Hawaiian Islands is part of a projected overview of the thrips fauna of this island chain. The first part of this overview included an extensive list of references to publications on Thysanoptera from Hawaii, and concerned the 99 species of the sub-order Terebrantia (Mound et al. 2016). A very high proportion of this fauna comprises taxa that have been introduced relatively recently, with less than 10% of Terebrantia species, and only five of the 18 species considered here, likely to be native to these islands.

The members of the Haplothripini share the following character states: antennae 8-segmented; head usually with a maxillary bridge clearly developed; prosternum with paired basantra well-developed; fore wings with a constriction medially; tergite IX of males with setal pair S2 short and stout; male sternite VIII usually without a pore plate. However, as is common in the diagnoses of other major groups of Thysanoptera - Phlaeothripidae, each of these character states is absent in one or more species of Haplothripini. One of the species included here, *Bamboosiella cingulata*, is included because it is similar in size and shape to species of *Haplothrips* and *Karnyothrips*, and is often taken with such species from grasses. However, the genus *Bamboosiella* is not considered a member of the Haplothripini (Okajima 2006; Mound and Minaei 2007). A further Haplothripini species, *Leptothrips mali* (Fitch), that is listed in the “Hawaiian all-species checklist database” (Bishop Museum 2002), is not included here because the record is indicated as based on a quarantine interception. There is no evidence that this North American predatory species has ever been found living on any of the Hawaiian Islands. Members of the genus *Leptothrips* are readily distinguished from almost all other Phlaeothripinae by the striate metanotum (Fig. 11). Full nomenclatural information about Thysanoptera is available on the web (ThripsWiki 2017).

## Results

### Key to *Haplothrips*-like species from Hawaiian Islands

**Table:** 

1	Maxillary stylets restricted to mouth cone, not retracted anterior to dorsal posterior margin of head (Fig. 3); prosternal basantra weakly developed [body strongly bicoloured, head, thorax and abdominal segments IX–X brown but segments I–VII and all tibiae yellow (Fig. 1); in grasses]	***Bamboosiella cingulata***
–	Maxillary stylets retracted into head, usually with an obvious maxillary bridge (Figs 4, 6, 7); prosternal basantra well-developed (Figs 8–10)	**2**
2	Antennal segment III with 3 sense cones	**3**
–	Antennal segment III with 1 or 2 sense cones	**5**
3	Fore femora with prominent tubercle on inner margin at base (Fig. 5); mouth cone not extending across prosternal basantra (cf. Figs 9–10) [in leaf galls on *Ficus* trees]	***Androthrips ramachandrai***
–	Fore femora without tubercle on inner margin at base; mouth cone long and pointed, extending across prosternal basantra (Fig. 8)	**4**
4	Pronotum with extensive and prominent sculpture lines (Fig. 6); antennal segment VIII almost as yellow as segment VII	***Dolichothrips franae***
–	Pronotum with few and weak sculpture lines (Fig. 7); antennal segment VIII brown in contrast to segment VII	***Dolichothrips indicus***
5	Fore tibia inner margin with sub-apical tubercle and small setal-bearing tubercle (Fig. 16); prosternal basantra longer than wide (Fig. 9)	***Podothrips lucasseni***
–	Fore tibia with no tubercle at inner apical margin; prosternal basantra usually wider than long (Fig. 10)	**6**
6	Antennal segment III sharply expanded into sub-basal ring distal to pedicel (Fig. 23)	***Priesneria doliicornis***
–	Antennal segment III tapering to base without a prominent ring (Figs 19–22)	**7**
7	Antennal segment III with only one sense cone	8
–	Antennal segment III with two sense cones	**11**
8	Wings reduced, either apterous or micropterous [fore tarsus without prominent tooth]	**9**
–	Wings fully developed and with duplicated cilia on distal hind margin (Fig. 17)	**10**
9	Apterous, ocelli absent; tergal wing-retaining setae small and straight; antennal segment IV with two sense cones; pronotal major setae pointed	***Apterygothrips remotus***
–	Micropterous, ocelli present; tergal wing-retaining setae long and sigmoid; antennal segment IV with three sense cones; pronotal setae capitate	***Karnyothrips longiceps***
10	Postocular setae capitate; antennal segment IV as yellow as III	***Haplothrips kurdjumovi***
–	Postocular setae acute; antennal segment IV brown in contrast to III	***Haplothrips graminis***
11	Fore wings broad, constricted medially but without duplicated cilia distally on posterior margin (Fig. 18); pronotal midlateral setae not developed	***Haplothrips robustus***
–	Fore wings present or absent, if present then with several duplicated cilia present distally on posterior margin (Fig. 17); pronotal midlateral setae usually well-developed	**12**
12	Body sharply bicoloured, head, thorax and abdominal segments IX–X brown, VIII variably shaded, tergites I–VII and all tibiae yellow (Fig. 2)	***Karnyothrips melaleucus***
–	Body largely brown	**13**
13	Setae S1 on tergite IX with apices capitate; fore tarsus with prominent recurved tooth (Fig. 12)	***Karnyothrips flavipes***
–	Setae S1 on tergite IX acute; fore tarsal tooth absent in females	**14**
14	Antennal segment IV with two sense cones	**15**
–	Antennal segment IV with three or four sense cones	**16**
15	Tergite IX setae shorter than tube; pronotal anteromarginal setae no longer than discal setae (Fig. 15); male sternite VIII with broad pore plate	***Haplothrips rosai***
–	Tergite IX setae longer than tube (Fig. 14); pronotal anteromarginal setae as long as anteroangular setae; male sternite VIII without pore plate	***Haplothrips davisi***
16	Antennal segment IV with three sense cones; legs yellow	***Haplothrips williamsi***
–	Antennal segment IV with four sense cones; legs mainly brown	**17**
17	Antennal segments III–V mainly yellow; postocular setae extending well beyond posterior margin of eye; tergite IX setae S1 acute and as long as the tube	***Haplothrips gowdeyi***
–	Antennal segments IV–V brown, III yellowish brown; postocular setae short, rarely extending to posterior margin of eye (Fig. 4); tergite IX setae S1 acute and scarcely as long as basal width of the tube	***Haplothrips leucanthemi***

**Figures 1–10.**
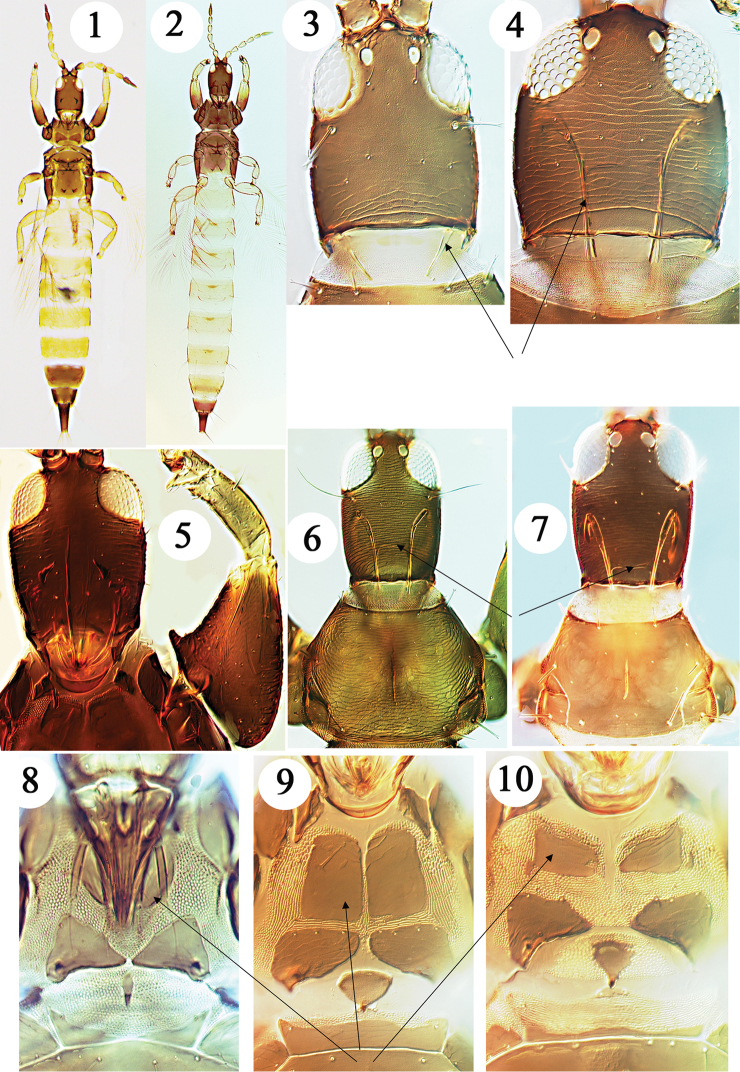
Haplothripine species from Hawaii. **1** *Bamboosiella cingulata* female **2** *Karnyothrips melaleucus* female **3** *B. cingulata* head **4** *Haplothrips leucanthemi* head (maxillary stylet indicated) **5** *Androthrips ramachandrai* female head and fore leg **6** *Dolichothrips franae* head and pronotum **7** *D. indicus* head and pronotum (maxillary bridge indicated) **8** *D. indicus* mouth cone and prosternites **9** *Podothrips lucasseni* prosternum **10** *Haplothrips davisi* prosternum (basisterna indicated by arrows .

#### Bamboosiella cingulata

Taxon classificationAnimaliaThysanopteraPhlaeothripidae

(Hood)

##### Remarks.

Described originally from Australia, with the synonym *sakimurai* from Oahu by Moulton (1937), this species is widespread throughout the tropics, and has been referred to frequently as *Antillothrips cingulata*. It is sometimes abundant, living in association with the leaves of tussocks of grass, but adults disperse and alight on other plants, and on Oahu have been recorded from lettuce leaves. The species is similar in size and colour to *Karnyothrips melaleucus*, with the head, thorax and abdominal apex dark brown but abdominal segments I–VII yellow. However, the maxillary stylets are short and restricted to the mouth cone (Fig. 3), and the prosternal basantra are only faintly indicated, whereas these prosternal sclerites are large in *Karnyothrips* species (cf Figs 8–10). The male remains unknown, and the genus is not regarded as a member of the Haplothripini (Okajima 2006).

#### Androthrips ramachandrai

Taxon classificationAnimaliaThysanopteraPhlaeothripidae

Karny

##### Remarks.

Described from India, this species has been reported from warmer parts of the Americas living in the leaf galls of *Gynaikothrips* species on *Ficus* trees (Boyd and Held 2006). In common with other members of the genus *Androthrips*, it has enlarged fore femora with a small tubercle at the base on the inner margin (Fig. 5). In contrast to some similar members of this genus the hind tibiae are dark brown, although antennal segments III–V are yellow. It is a predator of gall-inducing thrips (Melo et al. 2013), and in Australia has been found in galls induced by various species of Phlaeothripidae. On Hawaii, in the vicinity of Hilo, it has been found twice on the leaves of *Ficus*.

#### Apterygothrips remotus

Taxon classificationAnimaliaThysanopteraPhlaeothripidae

(Bianchi)

##### Remarks.

Described in the genus *Pseudocryptothrips*, but subsequently redescribed and transferred to *Apterygothrips* by Sakimura and Bianchi (1977), this species apparently remains known only from the original three females that were collected on Haleakala, Maui. These specimens were described as apterae, lacking ocelli, with the mesopresternum reduced to a pair of small lateral triangles, antennal segment III with one sense cone and segment IV with two, and all the major setae with their apices pointed.

#### Dolichothrips franae

Taxon classificationAnimaliaThysanopteraPhlaeothripidae

Mound & Okajima

##### Remarks.

Species of the genus *Dolichothrips* have the mouth cone unusually long and pointed (Fig. 8). *D. franae* has previously been known only from Oahu and Kauai, where it was living on the leaves of *Macaranga tanarius*. However, eight females have been studied that were collected in October 2014 from *Hibiscus tiliaceus* on Oahu, and a breeding population was found on Hawaii in May 2016 on the leaves of *Hibiscus brakenridgei*. This thrips species is known only from females, and it is likely to have been introduced to the Hawaiian Islands from somewhere in Southeast Asia (Mound and Okajima 2015). In contrast to the following species, the pronotum is extensively sculptured (Fig. 6).

**Figures 11–18.**
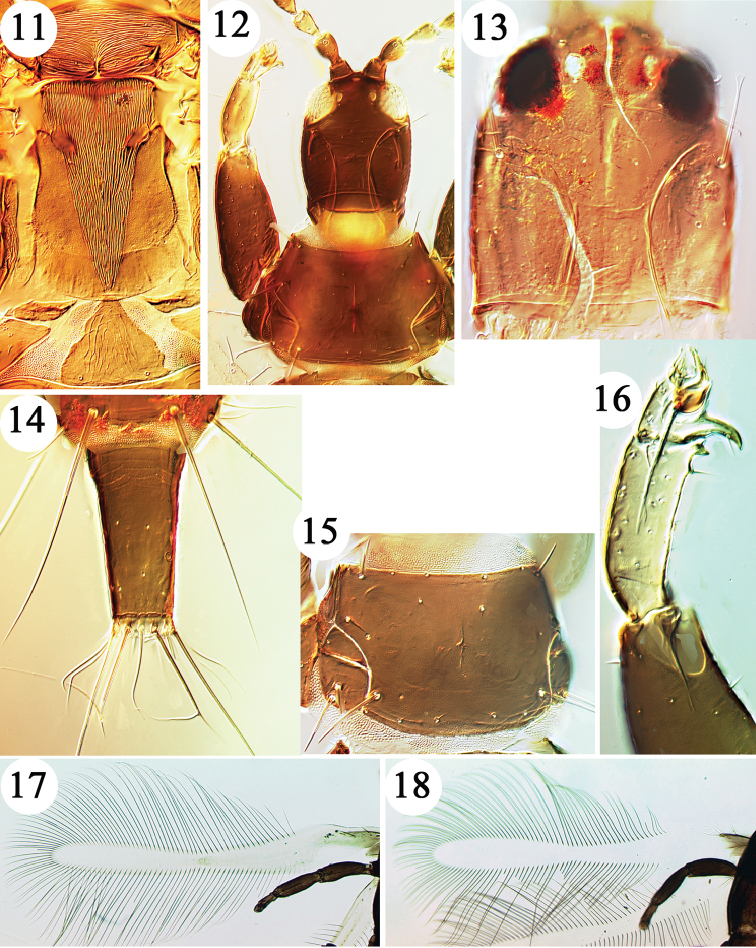
Haplothripine species from Hawaii. **11** *Leptothrips mali* meso and metanotum **12** *Karnyothrips flavipes* head and prothorax **13** *Haplothrips williamsi* head of holotype **14** *H. davisi* (*fissus* holotype tube and bifurcate anal setae) **15** *H. rosai* pronotum **16** *P. lucasseni* fore leg **17** *H. leucanthemi* fore wing with duplicated cilia **18** *Haplothrips robustus* fore wing without duplicated cilia.

#### Dolichothrips indicus

Taxon classificationAnimaliaThysanopteraPhlaeothripidae

(Hood)

##### Remarks.

Although described from India, this species has been found widely across the Pacific, including Japan, Taiwan, Guam, New Caledonia, and French Polynesia (Mound and Okajima 2015). On Hawaii it has been found established on the leaves of *Macaranga tanarius*, but is not recorded from any of the other Hawaiian Islands. It was recorded in the “Hawaiian all-species checklist database” (Bishop Museum 2002) under the synonymic name *Dolichothrips nesius* Stannard.

#### Haplothrips davisi

Taxon classificationAnimaliaThysanopteraPhlaeothripidae

Bianchi

Haplothrips davisi Bianchi, 1946: 503.
Haplothrips fissus Bianchi, 1947: 37. syn. n.

##### Remarks.

**Figures 19–24.**
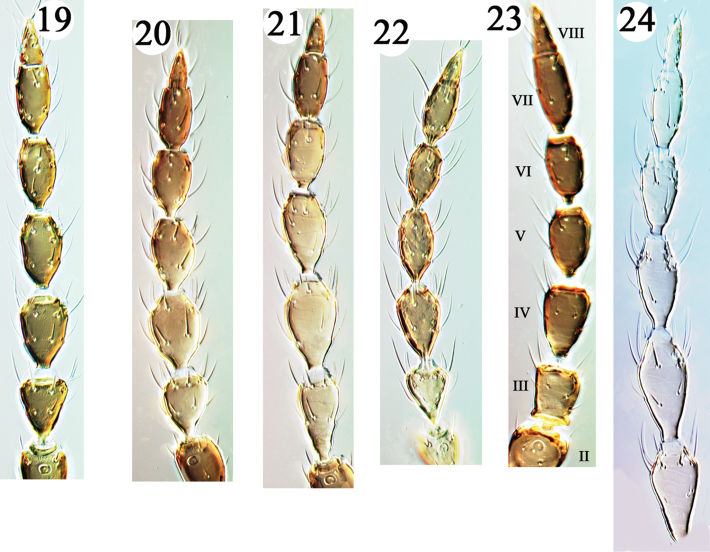
Haplothripine species from Hawaii, antennal segments II–VIII. **19** *H. davisi* **20** *H. gowdeyi* **21** *H. kurdjumovi* **22** *H. williamsi* **23** *P. doliicornis* (segments II–VIII indicated **24** *D. franae*.

The single female from which *fissus* was described is identical in structure with specimens *of davisi*, except that the two sub-median dorsal anal setae are bifurcate at a point approximately two-thirds along their length (Fig. 14). This condition is here interpreted as teratological, possibly resulting from damage as a pupa, although such aberrations are more commonly associated with damage to the anterior rather than the posterior end of phlaeothripid pupae. Described from nearly 20 specimens taken on Hawaii, both sexes of *davisi* were collected in July 2016 on Hawaii on Mauna Kea, from dead *Acacia koa* bearing lichens, and on Maui on Haleakala at approximately 2500m, from dead *Styphelia* with lichens. Although similar in general appearance to *rosai*, the two sense cones on each of antennal segments III and IV are stouter, segment VIII is broad at the base (Fig. 19), the major setae on tergite IX are longer than the tube (Fig. 14), and the male lacks a sternal pore plate. The pronotal anteromarginal and anteroangular pairs of setae are equally long, but they vary in length from scarcely three to more than five times as long as the pronotal discal setae. In the field, the adults could be mistaken for *Karnyothrips flavipes*, although the antennae and hind tibiae are dark brown, and in both sexes the fore tarsi lack a prominent tooth.

#### Haplothrips gowdeyi

Taxon classificationAnimaliaThysanopteraPhlaeothripidae

(Franklin)

##### Remarks.

Widely found in tropical countries around the world, this species is usually recognisable by the pale yellow colour of antennal segments III–VI that are in sharp contrast to the dark brown of the rest of the body. However, on the Hawaiian Islands there is a second species, *Haplothrips kurdjumovi*, that is quite similar in colour and general appearance. Despite the superficial similarities, *gowdeyi* has antennal segment III scarcely longer than wide, and thus almost sub-spherical (Fig. 20), and this segment bears two sense cones. These differences are discussed further below. Although reported as breeding in the inflorescences of grasses, *gowdeyi* also breeds in other flowers including Asteraceae (Mound and Wells 2015). Despite being widespread and common in tropical countries there have been no detailed studies on its biology.

#### Haplothrips graminis

Taxon classificationAnimaliaThysanopteraPhlaeothripidae

Hood

##### Remarks.

Described originally from Texas, and the synonymic species *fusca* Moulton from Oahu, this grass-living thrips is known to be widespread through Central America (Mound and Marullo 1996). In the Senckenberg Museum, Frankfurt, there are five males and five females identified by zur Strassen as this species that were collected on Lanai by Sakimura in August 1957 from *Paspalum conjugatum*, and in the USNM thrips collection, Beltsville, there are two slides of this species, bearing the synonymic name *fusca* Moulton, taken by Sakimura on Oahu in May, 1940, and on Molokai in May 1943. In the Department of Agriculture collection, Honolulu, there are specimens from *Pennisetum setaceum* that were collected in June 1990 on Hawaii. Currently no other records of this species from the Hawaiian Islands have been found.

#### Haplothrips kurdjumovi

Taxon classificationAnimaliaThysanopteraPhlaeothripidae

Karny

##### Remarks.

Described from Russia and widespread in Central Europe across Asia, this species is also introduced to North America and New Zealand (Minaei and Mound 2008). It appears to have been first identified from Hawaii in 2011, but in July 2016 considerable numbers of females were found at Volcano on *Carex* inflorescences as well as in the flowers of *Pyracantha* and *Rubus*. Moreover, two females were collected on Oahu at Palikea and the Mokuleia Trail. In the field it is easily mistaken for *gowdeyi*, because of the dark legs and almost clear yellow of antennal segments III–VI. Under a stereo microscope it is clear that antennal segment III is longer than wide and slightly asymmetric (Fig. 21), and on slide mounted specimens the presence of only a single sense cone on this segment can be confirmed. The species is reported to be a predator of mites and Lepidoptera eggs, but probably also feeds on floral tissues.

#### Haplothrips leucanthemi

Taxon classificationAnimaliaThysanopteraPhlaeothripidae

(Schrank)

##### Remarks.

This species, under the name *niger*, was reported from Maui by Sakimura (1990), based on specimens taken on Haleakala at an altitude of 6500–8500 ft from the grass *Anthoxanthum odoratum*, both in 1963 and 1977. However, the host plants of this thrips are not likely to be any species of Poaceae. Bisexual populations occur widely in Europe in the flowers of certain species of Asteraceae, particularly *Chrysanthemum leucanthemum*, the ox-eye daisy. A uni-sexual form that is commonly referred to as *H. niger*, occurs in the flowers of red clover, *Trifolium pratense*. Efforts by workers in Europe to distinguish the two forms morphometrically have been less than successful (Mound and Minaei 2007), and *niger* is currently considered a synonym. The species is unusual within the genus *Haplothrips* because of the short postocular setae on the head that do not extend to the posterior margin of the compound eyes (Fig. 4). Although not collected recently on the Hawaiian Islands, this European species is known from southern Australia, New Zealand, North America, Chile and Argentina (Hoddle et al. 2012).

#### Haplothrips rosai

Taxon classificationAnimaliaThysanopteraPhlaeothripidae

Bianchi

##### Remarks.

Described from three female and three male micropterae collected on Hawaii in the vicinity of Volcano, a single male of this species was taken in July 2016 on the lower slopes of Haleakala, Maui. In general appearance if is similar to *davisi*, but the pronotal anteromarginal pair of setae are no longer than the pronotal discal setae (Fig. 15), the major setae on tergite IX are shorter than the tube, and the male has a conspicuous transverse pore plate on the eighth sternite. Antennal segments III and IV each bear two rather weak and slender sense cones, and segment VIII is unusual in being constricted to the base. As with *davisi*, this apparently endemic species is probably mycophagous.

#### Haplothrips robustus

Taxon classificationAnimaliaThysanopteraPhlaeothripidae

Bagnall

Haplothrips robustus Bagnall, 1918: 209.
Haplothrips sesuvii Priesner, 1933: 363. syn. n.

##### Remarks.

This new synonymy is based on a comparison of a paralectotype of *sesuvii* from Java, on loan from the Senckenberg Museum, Frankfurt, with more than 50 specimens of *robustus* from across Australia, some of which were earlier compared with the holotype of this species in the Natural History Museum, London (Pitkin 1973; Mound and Minaei 2007). These two species were distinguished from each other by zur Strassen (1983), who provided seven sets of measurements, including wing width, maxillary bridge width and lengths of setae on tergites VIII and IX, each of which was larger in *robustus* than in *sesuvii*. However, although zur Strassen provided details of the type specimens of *sesuvii* that he studied, he did not indicate from what specimens of *robustus* his comparative measurements were taken. Measurements of the paralectotype of *sesuvii* fit well within the range of structural variation that occurs in *robustus* females from across Australia, and this variation is not unusual for a polyphagous, widespread species. Although known from Indonesia, Australia, New Hebrides, and Oahu, no male of this species is known, and the possibility exists that it may have been introduced from somewhere in Africa (Mound and Minaei 2007). In the Hawaiian Islands this thrips is recorded only from Oahu. The first record (Bianchi 1947), under the name *sesuvii*, was from *Sesuvium portulacastrum* [Aizoaceae] but specimens have been studied that were collected on Oahu by Sakimura at various dates between 1946 and 1970 from *Lipochaeta* [Asteraceae] and *Atriplex* [Chenopodiaceae]. Amongst the Hawaiian thrips fauna it is the only species with broad fore wings that are constricted medially but lack duplicated cilia on the distal posterior margin (Fig. 18).

#### Haplothrips williamsi

Taxon classificationAnimaliaThysanopteraPhlaeothripidae

Moulton

##### Remarks.

This species apparently remains known only from the original series of eight females taken at the western end of Hawaii in 1929. The original description was rather superficial, but the holotype and one paratype have now been re-examined. Based on these specimens, *williamsi* is one of the very few amongst the 240 species of *Haplothrips* that has two sense cones on antennal segment III but on segment IV has only three (instead of four) sense cones. The head (Fig. 13) and body are light brown with the tube darkest. The legs are almost clear yellow as is antennal segment III in contrast to the uniformly dark brown of segments IV–VIII. The antennal sense cones are rather stout with rounded apices. The maxillary stylets are retracted to the postocular setae, and one third of the head width apart with a distinct maxillary bridge. The fore tarsus has a slight thickening at the inner apex that might be interpreted as a particularly minute tooth. The metanotum lacks sculpture medially, and the median major setae are finely pointed. In contrast, most of the major setae are capitate including S1 on tergite VIII, but S2 on VIII and all three pairs on IX are finely acute and longer than the tube. This species shows interesting similarities to the endemic species *davisi* and *rosai*, in the the fore tarsus and the conical form of antennal segment III, also the elongate acute setae on tergite IX.

#### Karnyothrips flavipes

Taxon classificationAnimaliaThysanopteraPhlaeothripidae

(Jones)

##### Remarks.

This is one of the most commonly collected Phlaeothripidae on the Hawaiian Islands, where it has been taken widely, living on dead branches and twigs. Although common and widespread on the islands, it is rarely found in large numbers, a characteristic that agrees with its habits as a predator of other small arthropods (Jaramilo et al. 2010). Small and dark brown, with a slightly elongate head (Fig. 12), it is usually recognisable from the slightly yellow colour of antennal segment III, and the hind tibiae that are yellowish on at least the distal half. The number of antennal sense cones varies in this species, with segment IV bearing either three or four, although segment III consistently bears two sense cones (Okajima 2006). The fore tarsus bears a prominent curved tooth at the inner apex (Fig. 12), and the major setae S1 and S2 on tergite VIII are capitate, as is S1 on the ninth tergite.

#### Karnyothrips longiceps

Taxon classificationAnimaliaThysanopteraPhlaeothripidae

(Hood)

##### Remarks.

There is a slide of this species in the USNM collection, Beltsville, taken in quarantine from Hawaii at San Pedro, California in March, 1969, and this is possibly the basis for *longiceps* being listed from Hawaii. However, two micropterous females of this species were collected from dead *Acacia koa* on Hawaii in July 2016, one at Hakalau, Mauna Kea, the other at Kipuka Ki, Hawaii Volcanoes National Park. Described originally from Illinois, USA, it appears to be widespread south through Central America to Chile and Peru (Mound and Marullo 1996). It is the same size and colour as *flavipes*, but the fore tarsal tooth is minute and the hind tibiae almost completely yellow. Apart from the presence of two pairs of sigmoid setae on the tergites, *longiceps* could equally well be placed in *Apterygothrips*, since these two genera are not clearly distinguished by the available generic diagnoses.

#### Karnyothrips melaleucus

Taxon classificationAnimaliaThysanopteraPhlaeothripidae

(Bagnall)

##### Remarks.

Although not taken as frequently as *K. flavipes*, this worldwide bicoloured predatory thrips occurs widely on the Hawaiian Islands. It is found more frequently on grasses than on dead branches, and is similar in general appearance to *Bamboosiella cingulata* (Figs 1–2). However, in contrast to that species the maxillary stylets are deeply retracted and close together medially in the head, the prosternal basantra are well-sclerotised, the mid and hind legs are clear yellow, and tergite VIII is yellow at least in part.

#### Podothrips lucasseni

Taxon classificationAnimaliaThysanopteraPhlaeothripidae

(Krüger)

##### Remarks.

Described from Java, with one synonym from Hawaii and another from Thailand, this species is widespread in tropical Asia from India to northern Australia (Ritchie 1974), but specimens have also been studied from South Africa and Madagascar. It is a predatory species on coccoids living on Poaceae, and is particularly associated with sugar cane. A brown species with clear yellow tibiae, it is one of the species of *Podothrips* in which the fore wings lack duplicated cilia. The fore femora inner margin is slightly rugose, and in large individuals is sometimes rather swollen. But it is unusual within the genus in having a pair of small triangular sclerites lateral to the pelta that represents the first abdominal tergite. As in other members of the genus *Podothrips*, the prosternal basantra are longer than wide (Fig. 9), and there is a small tubercle on the inner apex of the fore tibiae (Fig. 16).

#### Priesneria doliicornis

Taxon classificationAnimaliaThysanopteraPhlaeothripidae

(Bianchi)

##### Remarks.

Described in *Karnyothrips* but transferred by Pitkin (1973) to the genus *Priesneria* because of the curious third antennal segment (Fig. 23), the relationships of this species remain in some doubt. Two species with similar antennae are described in the genus *Karnyothrips* from southern Japan (Okajima 2006), of which *antennalis* has one sense cone on segment III and three on segment IV, whereas *inflatus* is particularly similar to *doliicornis* in having two on both III and IV. A third species with similar antennal structure has been described in *Karnyothrips* from southern China (Wang et al. 2013), but that has two sense cones on segment III and four on IV. The curious shape of antennal segment III, and the number of sense cones on III and IV, may not be good indicators of relationships in the *Karnyothrips* group. Bianchi described *doliicornis* from 10 females collected on Hawaii, of which nine were micropterous and one macropterous. Two micropterous females were collected in July 2016 at sites near Volcano, Hawaii, one on dead branches of *Metrosideros*, and one on dead branches of *Dodonaea*.

## Supplementary Material

XML Treatment for Bamboosiella cingulata

XML Treatment for Androthrips ramachandrai

XML Treatment for Apterygothrips remotus

XML Treatment for Dolichothrips franae

XML Treatment for Dolichothrips indicus

XML Treatment for Haplothrips davisi

XML Treatment for Haplothrips gowdeyi

XML Treatment for Haplothrips graminis

XML Treatment for Haplothrips kurdjumovi

XML Treatment for Haplothrips leucanthemi

XML Treatment for Haplothrips rosai

XML Treatment for Haplothrips robustus

XML Treatment for Haplothrips williamsi

XML Treatment for Karnyothrips flavipes

XML Treatment for Karnyothrips longiceps

XML Treatment for Karnyothrips melaleucus

XML Treatment for Podothrips lucasseni

XML Treatment for Priesneria doliicornis
